# Reciprocal Effect of Copper and Iron Regulation on the Proteome of *Synechocystis* sp. PCC 6803

**DOI:** 10.3389/fbioe.2021.673402

**Published:** 2021-05-10

**Authors:** Zhang-He Zhen, Song Qin, Qing-Min Ren, Yu Wang, Yu-Ying Ma, Yin-Chu Wang

**Affiliations:** ^1^Yantai Institute of Coastal Zone Research, Chinese Academy of Sciences, Yantai, China; ^2^University of Chinese Academy of Sciences, Beijing, China; ^3^Center for Ocean Mega-Science, Chinese Academy of Sciences, Qingdao, China

**Keywords:** cyanobacteria, copper, iron, proteome, deduction

## Abstract

Cyanobacteria can acclimate to changing copper and iron concentrations in the environment via metal homeostasis, but a general mechanism for interpreting their dynamic relationships is sparse. In this study, we assessed growth and chlorophyll fluorescence of *Synechocystis* sp. PCC 6803 and investigated proteomic responses to copper and iron deductions. Results showed that copper and iron exerted reciprocal effect on the growth and photosynthesis of *Synechocystis* sp. PCC 6803 at combinations of different concentrations. And some proteins involved in the uptake of copper and iron and the photosynthetic electron transport system exhibit Cu–Fe proteomic association. The protein abundance under copper and iron deduction affected the photosynthetic electronic activity of *Synechocystis* sp. PCC 6803 and eventually affected the growth and photosynthesis. Based on these results, we hypothesize that the Cu–Fe proteomic association of *Synechocystis* sp. PCC 6803 can be elucidated via the uptake system of outer membrane-periplasmic space-inner plasma membrane-thylakoid membrane, and this association is mainly required to maintain electron transfer. This study provides a broader view regarding the proteomic association between Cu and Fe in cyanobacteria, which will shed light on the role of these two metal elements in cyanobacterial energy metabolism and biomass accumulation.

## Introduction

Cyanobacterial metal homeostasis is important for the metal ion-driven photosynthetic machinery, rendering metals the limiting factors for cyanobacteria growth (Huertas et al., 2014; Chandra and Kang, 2016). Both copper and iron are essential metal elements for cyanobacteria. Iron, important trace metal element in the cyanobacterial thylakoid membrane (TM), has been shown to be involved in photosynthetic electron transfer, respiratory electron transfer, light-harvesting pigment synthesis, and various other important metabolic processes (Öquist, 1974; Sedwick et al., 1999). Copper is also an essential micronutrient that plays crucial roles in metal homeostasis and normal plant metabolism during photosynthesis and respiration (Yruela et al., 2000; Bernal et al., 2004).

The mechanism via which copper and iron functioned together has undergone environmental changes during the evolution of cyanobacteria. Notably after the Great Oxidation Event 2.35 billion years ago, the soluble copper content increased significantly, whereas the bioavailability of iron became limited in aquatic environments (Holland, 2002; Anbar et al., 2007). Therefore, cyanobacteria are believed to have gradually evolved different metal-binding proteins to acclimate to the changing environment, and some copper-containing proteins have functionally replaced iron-related proteins, reducing the effect of iron restriction on cell growth (De la Cerda et al., 2007). The appearance of plastocyanin in cyanobacteria probably conferred a selective advantage in iron-limited ecosystems, such as in more oxidizing environments, by releasing O_2_ via oxygenic photosynthesis (Molina-Heredia et al., 2002; Peers and Price, 2006). These imply an evolutionary association between copper and iron in cyanobacteria.

Cyanobacteria have also developed physiological regulatory mechanisms to acclimate to environmental changes in copper and iron levels. Intracellular copper and iron content must be balanced via mechanisms depending on their aquatic bioavailability. These two essential metal elements work together in some processes. Copper metalloenzymes and iron metalloenzymes can participate in diverse cellular processes, such as energy transduction and oxidative stress response (Huertas et al., 2014), and sometimes Cu-binding and Fe-binding proteins can function interchangeably. For instance, it is well known that the iron-binding cytochrome *c*_6_ (Cyt *c*_6_) replaces the copper-binding plastocyanin in the absence of copper (Molina-Heredia et al., 2002). In addition, iron and copper act synergistically have unraveled the relationship between iron and copper homeostasis in cyanobacteria (Nicolaisen et al., 2010). On one hand, a cuproenzyme is involved in iron mobilization (Rensing and Grass, 2003; Rademacher and Masepohl, 2012; Huertas et al., 2014). On the other hand, the periplasmic iron-binding protein, FutA2, is related to copper import in *Synechocystis* sp. PCC 6803 (hereinafter *Synechocystis* 6803) (Waldron et al., 2007); a TonB-dependent iron transporter (*iacT*, All4026), located at the outer membrane (OM), affected the rate of copper transport (Nicolaisen et al., 2010). Overall, several studies have elucidated that copper and iron function together and compensate for each other’s levels in cyanobacteria.

Currently, reports regarding Cu–Fe association and its association mechanism are scarce. In mutant strains of *Anabaena* sp. PCC 7120, Nicolaisen et al. (2010) observed that the iron and copper content is affected by the TonB-dependent iron transporter, which revealed the intracellular homeostasis of copper and iron, although the specific mechanism via which homeostasis is maintained has not been reported. In addition, using proteomics, Castielli et al. (2009) analyzed the protein associations during copper and iron deduction in *Synechocystis* 6803 and confirmed the substitution reaction of Plastocyanin and Cytochrome *c* on the electron transfer chain; however, they did not comprehensively analyze other copper-related and iron-related proteins. Therefore, a general mechanism for interpreting their dynamic relationships is sparse, especially when one or both are limited or lacking. Fortunately, many detailed reports regarding the effects of individual iron or copper deduction stress on cyanobacteria are now available. On one hand, many studies regarding cyanobacterial iron deduction stress have showed that iron deficiency can induce remodulation of photosystem complexes (Michel and Pistorius, 2004; Latifi et al., 2005; Allen et al., 2008), and that the expression of iron stress-induced chlorophyll binding protein A (IsiA) (Burnap et al., 1993), iron transporters (Shcolnick et al., 2009), and alternative redox vectors were up-regulated (Wood, 1978; Sandmann, 1985). On the other hand, copper deduction has been shown to reduce synthesis of plastoquinone and disintegration of thylakoid membranes (Hutber et al., 1977; Guikema and Sherman, 1983; Ivanov et al., 2000; Sandström et al., 2002), and upregulate proteins involved in the main metabolic pathways, such as C and N fixation and carbohydrate metabolism (Angeleri et al., 2019). Based on the extensive influence of iron and copper on cell metabolism (Meisch and Bielig, 1975; Verma and Singh, 1990; Shavyrina et al., 2001; Pehlivan, 2018), we speculated that the association of Cu–Fe-related proteins may not only be limited to the electron transport chain, but may have a broader physiological impact to cyanobacteria.

In this study, the growth pattern of *Synechocystis* 6803 in response to the given copper-iron combination was examined, then followed by comparative proteomic analyses to reveal the proteins associated with copper and iron stress. We anticipate that our study would provide a theoretical basis for understanding the relationship between copper and iron in cyanobacteria at the protein level and shed light on the role of these two metal elements in energy metabolism and biomass accumulation of cyanobacteria.

## Materials and Methods

### Metal-Deduction Treatments and Growth Curve Measurements

To deplete the stored copper and iron in the cells to meet the set concentration gradients, the *Synechocystis* 6803 were cultured in BG11 medium (with copper sulfate and ferric ammonium citrate removed from the normal recipe) lacking Fe and Cu for 5 days under normal conditions as mentioned below: light intensity = 50 μmol photons m^–2^ s^–1^, light/dark period = 12 h/12 h, and supply of filtered air bubbled at 30°C (Rippka et al., 1979; Singh et al., 2003; Georg et al., 2017). The method of determining the abundance of copper and iron refers to other reports (Varga et al., 1999; Li et al., 2011).

To determine the optimum iron and copper concentrations for the growth of *Synechocystis* 6803, the strain was transferred to normal BG11 medium supplemented with 25 different combinations of copper and iron concentrations for acclimatation and maintained in exponential growth phase for 3 days in advance. Copper and iron concentrations were set to form a 5 × 5 matrix: copper C1−C5 (0, 112.5, 225, 337.5, and 450 nM) and iron F1−F5 (0, 6.25, 12.5, 18.75, and 25 μM). The matrix covers the optimal Cu/Fe concentration combination for *Synechocystis* growth via beforehand repeated exploratory experiments. The copper concentrations were set below the copper toxic concentration (0.5 μM) to *Synechocystis* 6803 (Cheloni et al., 2019). And the iron concentrations were set to coordinate with the copper conditions to balance the coverage and precision. The Cu/Fe concentrations of the 25 cultures and their designations were based on the combinations of the two series. For instance, group C2F4 contained 112.5 nM copper (C2) and 18.75 μM iron (F4). Then, cultures were diluted to equal cell densities [OD_730_ = 0.04 in a 96-well plate (200 μL) using a microplate reader] and were transferred to new medium (with no change in Cu/Fe concentrations and incubation conditions). Finally, the cell densities were measured after every 24 h in a 96-well plate (200 μl) using a microplate reader (Shanpu SuPerMax 3100, Shanghai, China) (the standard curve of optical densities measured with the microplate reader and normal spectrophotometer at 730 nm of *Synechocystis* 6803 was shown in Supplementary Figure 1). Three replicates were performed for each group.

### Chlorophyll Fluorescence Measurements

Chlorophyll fluorescence of the 25 cultures (each group with three replicates) were measured using Diving-PAM (WALZ, Germany) to analyze how Cu/Fe concentrations affected photosynthesis in the strain. During logarithmic growth phase (120 h), cultures were acclimated in dark for at least 15 min, and *F*_*o*_, *F*_*m*_, *F*_*m*_ parameters were measured under actinic light (50 μmol photons m^–2^ s^–1^) after applying a saturating pulse (3,577 μmol photons m^–2^ s^–1^, 800 ms) (Xu et al., 2012). The maximum quantum yield of PSII (*F*_*v*_/*F*_*m*_), the actual effective quantum yield of PSII [Y(II)], and the relative electron transfer rate (rETR) were calculated according to Maxwell (Maxwell and Johnson, 2000).

### Protein Extraction and TMT Tagging

With the fastest growing cultures (the F4C4 group, see section “Physiological response to copper and iron deduction” in Result) set as the control group, the Fe-deduction group (i.e., F1C4), Cu-deduction group (i.e., F4C1), and Cu–Fe dual deduction group (i.e., F1C1) were set as the experimental groups. Three replicates were performed for each group. These cultures have the same culture conditions and growth status as the cultures used for growth curve measurement and were investigated using TMT quantitative proteomics to determine the effect of Cu/Fe deductions on *Synechocystis* 6803 protein levels. Cells in logarithmic growth phase were collected for proteomic determination after 120 h of metal-deduction.

Proteins were extracted from cyanobacteria using the SDT [4% w/v sodium dodecul sulfate (SDS), 100 mM Tris/HCl pH 7.6, 0.1 M dithiothreitol (DTT)] cleavage method (Wiśniewski et al., 2009), and total protein levels were quantified using the bicinchoninic acid assay (BCA) method. Each sample was trypsinized using the filter-aided proteome preparation (FASP) method and the peptides were quantified (OD_280_) (Wiśniewski et al., 2009). The peptides (100 μg) of each sample were labeled according to the instructions of the TMT labeling kit (Thermo, United States).

### LC MS/MS Data Collection and Analysis

The TMT-labeled peptides of each group were mixed in equal amounts and fractionated using the high pH reverse phase peptide fractionation kit (Thermo, United States). Each fractionated sample was separated using high performance liquid chromatography (HPLC) (Thermo scientific, United States). Buffer A consisted of 0.1% formic acid solution and buffer B contained 0.1% formic acid-84% acetonitrile solution. The column was equilibrated with 95% buffer A. Samples were separated using a loading column (nanoViper C18, Thermo Scientific Acclaim PepMap100, 100 μm × 2 cm) and an analytical column (Thermo Scientific EASY column, 10 cm, ID75 μm, 3 μm, C18-A2). The flow rate was set to 300 nL/min.

The separated samples were analyzed using Q-Exactive MS under positive ion mode. The scanning range of the precursor ion was 300−1800 *m/z*, AGC (automatic gain control) target was 1 × 10^–6^, maximum IT was 50 ms, and dynamic exclusion time was 60.0 s. Several 20 fragment maps (MS2 scan, MS2 activation type: HCD) were collected after each full scan. The isolation window was set to 2 *m/z*. Normalized collision energy was 30 eV and underfill was 0.1%.

The MS data was identified and quantitatively analyzed using Mascot ver. 2.2 (Matrix Science) and Proteome Discoverer 1.4 (Thermo, United States). The maximum missed cleavage was set to 2; peptide mass tolerance and fragment mass tolerance was set to ±20 ppm and 0.1 Da, respectively. Peptide false discovery rate (FDR) was set at < 0.01. Protein quantification was performed based on the median of only the unique peptides of the protein. The mass spectrometry proteomics data have been deposited to the ProteomeXchange Consortium via the PRIDE (Perez-Riverol et al., 2019) partner repository with the dataset identifier PXD024873.”

### Data Analysis

SPSS ver. 19.0 was used to perform parametric one-way analysis of variance (ANOVA) for analyzing the differences in the growth rate, *F*_*v*_/*F*_*m*_, Y(II), and rETR values of the cultures among different groups.

Differentially expressed proteins were filtered using a fold change ≥ 1.2 and ≤0.83, and *P*-value < 0.05 (paired *t*-test or one-way ANOVA) (Cox and Mann, 2008). Blast2GO ver. 5.2 (BioBam, Spain) was used to annotate Gene Ontology (GO) function entries for all identified proteins, and Kyoto Encyclopedia of Genes and Genomes (KEGG) Automatic Annotation Server (KAAS) was used to allocate the differentially expressed proteins to the biological pathways. GO function and each KEGG pathway protein were analyzed for the significance of enrichment using Fisher’s exact test.

The paired *t*-tests were performed on the corrected ratios (the intensity of the fragmented tag in a sample to the intensity of the fragmented tag in the control sample) to identify the proteins and their proteomic associations after Fe or Cu deductions. The Cochran–Mantel–Haenszel tests for repeated 2 × 2 tests of independence were performed to identify the proteins under the conditional association of copper and iron (Rayner and Rippon, 2018). All tests were performed and verified using SPSS ver. 19.0.

## Results

### Physiological Response to Copper and Iron Deduction

**FIGURE 1:**
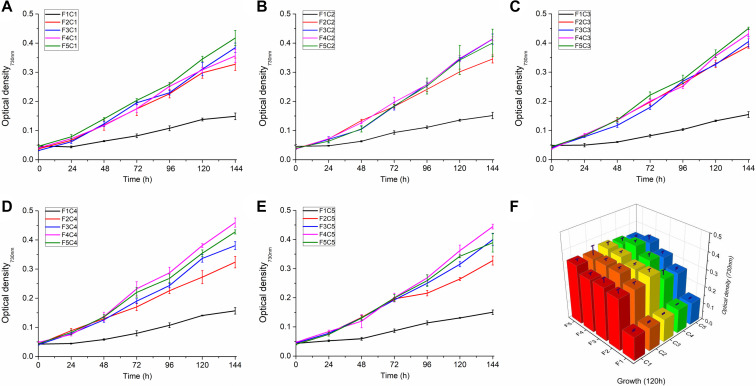
Growth of *Synechocystis* sp. PCC 6803 under different Cu and Fe concentrations. **(A–E)** The growth curve of *Synechocystis* sp. PCC 6803 in the presence of different combinations of copper and iron concentrations; **(F)** Growth of *Synechocystis* sp. PCC 6803 at 120 h. C1∼C5 indicate Cu concentrations of 0, 112.5, 225, 337.5, and 450 nM, respectively; F1∼F5 indicate Fe concentrations of 0, 6.25, 12.5, 18.75, and 25 μM, respectively.

The growth rates of 25 groups of *Synechocystis* 6803 under different culture conditions in terms of combinations of copper and iron concentrations were continuously monitored for 7 days (Figure 1). As shown in Figure 1, the increase of iron concentration from F1 to F5 tends to promote cell growth (Figures 1A–E), and the growth rates of F1 groups were significantly lower (*P* < 0.05). The change of copper concentrations has little effect on cell growth rate in the F1–F5 series, but the change of copper concentration affected the optimal iron concentration for growth. The fastest growth rate was observed in the F4C4 group (OD_730_ = 0.404), followed by that in the F5C3 group (OD_730_ = 0.393) (Figure 1F).

**FIGURE 2:**
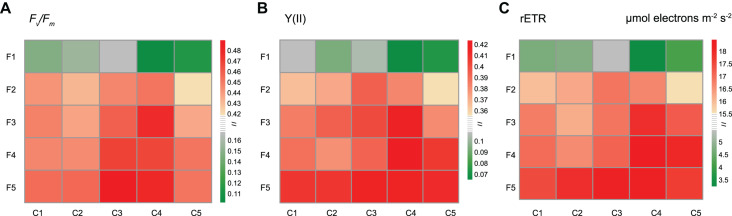
Effects of different copper and iron concentrations on the chlorophyll fluorescence of *Synechocystis* sp. PCC 6803. **(A)** F_v_/F_m_ (the maximum quantum yield of PSII); **(B)** Y(II) (the actual effective quantum yield of PSII); **(C)** rETR (the relative electron transfer rate).

As shown in Figure 2, three parameters related to chlorophyll fluorescence were measured: *F*_*v*_/*F*_*m*_ (the maximum quantum yield of PSII), Y(II) (the actual effective quantum yield of PSII) and rETR (the relative electron transfer rate). In the C1−C3 series, the fluorescence parameters increased with Fe concentrations (from F1 to F5 series) and peaked in the F5 group. However, for C4 and C5 series, the highest value was not observed in the F5 group. The maximum values of rETR and Y(II) were observed in the F4C4 group, while the maximum value of *F*_*v*_/*F*_*m*_ was observed in the F5C3 group.

### General Proteomic Analyses

The effects of copper deduction, iron deduction, and copper-iron deduction on the protein profile of *Synechocystis* 6803 were investigated using TMT quantitative proteomics technology. In total, 1,555 proteins were identified based on 8350 peptides and 8197 unique peptides identified in *Synechocystis* 6803; proteins with expression ≥ 1.2 times or ≤0.83 times that of the control and *p*-value < 0.05 were considered significant.

**FIGURE 3:**
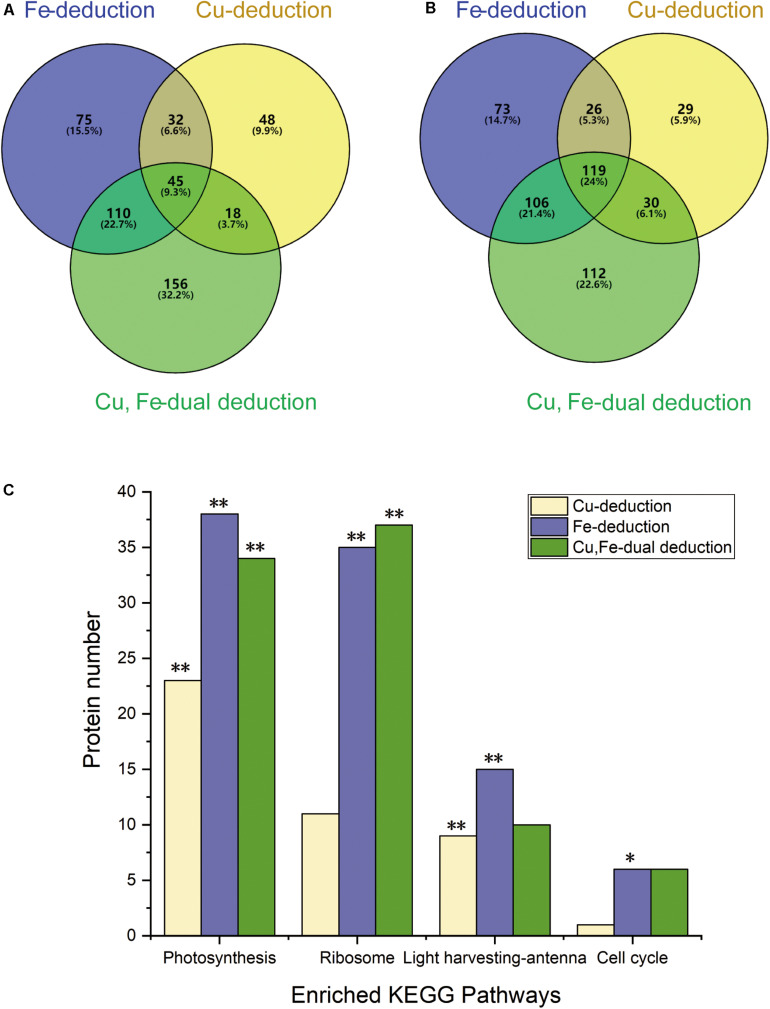
Venn diagrams showing differential protein abundances and enriched KEGG pathways. **(A)** Up-regulated proteins (≥1.2-fold); **(B)** down-regulated proteins (≤0.83-fold); **(C)** **P < 0.01, *P < 0.05.

Venn diagrams show (Figures 3A,B) that the number of differentially expressed proteins induced by dual deduction of copper and iron was the largest (up, 329; down, 367), followed by those induced by iron deduction (up, 262; down, 324), while copper deduction induced the least number of differentially expressed proteins (up, 143; down 204).

The GO functional analysis of all the differentially expressed proteins yielded 139 GO functional annotation entries (*P* < 0.05) in the Fe-deduction group, 52 GO functional annotation entries (*P* < 0.05) in the Cu-deduction group and 87 GO functional annotation entries (*P* < 0.05) in the Cu, Fe dual deduction group. These proteins were further categorized into three groups: biological processes, cellular components, and molecular functions based on functional differences. Table 1 lists the first eight groups of most significant annotations. Some photosynthesis-related and non-photosynthesis-related differentially expressed proteins present in these annotations have been listed in Supplementary Tables 1, 2, respectively.

**TABLE 1:** Enriched Gene Ontology (GO) term.

**Condition**	**Go Term**	**Protein number**	***P*-value**	**Category**
Fe-deduction	Photosynthetic membrane	72	2.57E-13	CC
	Thylakoid part	74	3.43E-13	CC
	Thylakoid	75	3.91E-13	CC
	Thylakoid membrane	67	3.88E-12	CC
	Protein-containing complex	111	6.01E-12	CC
	Organelle part	55	4.3E-11	CC
	Membrane protein complex	57	1.5E-10	CC
	Light-harvesting complex	19	9.56E-09	CC
	Photosynthesis	58	1.85E-09	BP
	Protein-chromophore linkage	15	4.99E-06	BP
	Cellular protein metabolic process	72	0.000443	BP
	Protein metabolic process	78	0.000815	BP
	Electron transport chain	16	0.000999	BP
	Establishment of protein localization	18	0.001214	BP
	Protein localization	18	0.001214	BP
	Macromolecule localization	18	0.001214	BP
	Electron transfer activity	25	3.95E-06	MF
	Tetrapyrrole binding	18	9.64E-05	MF
	Structural constituent of ribosome	35	0.000153	MF
	Structural molecule activity	36	0.000249	MF
	Heme binding	11	0.000797	MF
	rRNA binding	24	0.000804	MF
	ATP-dependent peptidase activity	8	0.008742	MF
	Chlorophyll binding	6	0.014444	MF
Cu-deduction	Cell envelope	38	3.37E-09	CC
	Envelope	38	4.26E-08	CC
	Periplasmic space	35	5.6E-08	CC
	Outer membrane-bounded periplasmic space	33	2.1E-07	CC
	Thylakoid	45	8.89E-07	CC
	Thylakoid part	43	3.76E-06	CC
	Membrane	101	8.04E-06	CC
	Photosynthetic membrane	40	2.19E-05	CC
	Protein-chromophore linkage	11	9.73E-05	BP
	Photosynthesis	33	0.000223	BP
	Carbohydrate transport	5	0.007985	BP
	Lipid catabolic process	3	0.011425	BP
	Organic substance transport	14	0.014585	BP
	Localization	25	0.018939	BP
	Transport	24	0.021303	BP
	Establishment of localization	24	0.021303	BP
	Electron transfer activity	14	0.005798	MF
	Siderophore transmembrane transporter activity	3	0.011425	MF
	Siderophore uptake transmembrane transporter activity	3	0.011425	MF
	Cofactor transmembrane transporter activity	3	0.011425	MF
	Wide pore channel activity	5	0.017382	MF
	Porin activity	5	0.017382	MF
	Tetrapyrrole binding	10	0.020033	MF
	Iron ion binding	10	0.020033	MF
Cu, Fe-dual deduction	Cell envelope	56	1.34E-09	CC
	Envelope	59	1.61E-09	CC
	Periplasmic space	53	8.23E-09	CC
	Outer membrane-bounded periplasmic space	51	1.01E-08	CC
	Protein-containing complex	112	6.48E-07	CC
	Thylakoid membrane	63	3.37E-06	CC
	Photosynthetic membrane	66	4.8E-06	CC
	Thylakoid	69	6.53E-06	CC
	Photosynthesis	55	6.47E-05	BP
	Protein metabolic process	91	0.000299	BP
	Cellular protein metabolic process	81	0.00092	BP
	Peptide metabolic process	59	0.001128	BP
	Translation	56	0.001475	BP
	Peptide biosynthetic process	56	0.001475	BP
	Carbohydrate transport	7	0.003874	BP
	Homeostatic process	18	0.00607	BP
	Structural molecule activity	40	0.000329	MF
	Structural constituent of ribosome	38	0.000453	MF
	Porin activity	8	0.001747	MF
	Wide pore channel activity	8	0.001747	MF
	ATP-dependent peptidase activity	9	0.004681	MF
	Iron ion binding	17	0.004966	MF
	Electron transfer activity	22	0.00597	MF
	Enzyme binding	6	0.008584	MF

*BP, biological process; MF, molecular function; CC, cellular component.*

In all three deduction groups, various cellular components of the thylakoid and photosynthetic membrane changed significantly. For example, the significantly down-regulated proteins (*P* < 0.05) included the chloroplast import-associated channel IAP75, membrane protein PilM and membrane-associated protein Slr1513, while the significantly up-regulated proteins included cyanobacterial orthologs of chloroplast proteins Ycf35 and Ycf23 (*P* < 0.05) (Supplementary Table 2); Photosynthesis was the most predominant biological process (BP) under all three conditions (Table 1), and various proteins involved in photosynthesis were significantly down-regulated, including photosynthesis assembly proteins (Slr0147, Slr0149, and Slr0151) and proteins related to phycobilisomes, PSII, PSI, Cytochrome *b*_6_/*f* complex, photosynthetic electron transporter, and ATP synthase (Supplementary Table 1). However, individual proteins were up-regulated in response to certain conditions. For example, Cytochrome *c*_6_ (18.06-fold) and Plastocyanin (1.44-fold) were significantly up-regulated in the Cu-deduction and Fe-deduction groups, respectively. Psb28 (Fe-deduction group: 1.54-fold; Cu–Fe dual deduction group: 2.51-fold) and IsiA (Fe-deduction group: 4.41-fold; Cu-Fe dual deduction group: 4.52-fold) were only up-regulated in response to iron deduction, but did not respond significantly to copper deduction. In addition, protein metabolism was one of the significantly enriched biological processes in the Fe-deduction group (Supplementary Table 1). Various ribosome-related proteins participated in this process (Supplementary Table 2), most of which were significantly down-regulated and few were significantly up-regulated. In the Cu-deduction group carbohydrate transport, lipid catabolic process, and organic substance transport (*P* < 0.05) were the significantly affected biological processes, which affected the expression of several metabolic enzymes, such as carbohydrate metabolizing enzymes, including ATP-dependent 6-phosphofructokinase (2.27-fold), Alpha-1,4 glucan phosphorylase (1.32-fold), and Aconitate hydratase B (1.59-fold), and various other carbon or nitrogen metabolism-associated proteins, including Biotin carboxylase (2.07-fold), Nitrate reductase (2.01-fold), Aminopeptidase (1.69-fold), Ribose phosphate isomerase B (1.46-fold), Glucose-6-phosphate 1-dehydrogenase (1.31-fold), and Glutamine synthetase (1.24-fold) (Supplementary Table 2). The molecular functions differed significantly in these three experimental groups (Table 1). Iron deduction and copper deduction alone affected electron transfer activity; however, in the group of Cu, Fe dual-deduction, the main molecular functions affected included structural molecule activity and structural constituent of ribosome (Table 1).

KEGG analysis of differentially expressed proteins showed that these proteins were mainly concentrated in the four signaling pathways of photosynthesis, photosynthetic-antenna proteins, ribosomes, and the cell cycle under the three deduction conditions (Figure 3C). Photosynthesis and photosynthetic-antenna proteins were highly enriched (*P* < 0.01) in the Cu-deduction and Fe-deduction groups (*P* < 0.01), while ribosomal proteins were highly enriched (*P* < 0.01) in the Cu, Fe dual deduction group, in addition to photosynthetic proteins. Furthermore, the cell cycle signaling pathway was also significantly enriched in the Fe-deduction group (*P* < 0.05) (Figure 3C).

### Cu–Fe Proteomic Associations

Cochran–Mantel–Haenszel tests were performed to analyze whether protein expression was affected by the copper-iron reciprocal effect. We observed that the expression of ten proteins showed significant copper-iron proteomic association (*P* < 0.05) (Table 2), among which five were porins, two were iron transporters, and the remaining three were photosynthetic electron transport-related proteins.

**TABLE 2:** Reciprocal effect of copper and iron on protein expression.

**Protein**	**Gene**	***P*-value**
Slr1908	*slr1908*	0.00005
Slr1841	*slr1841*	0.00011
Flavodoxin	*isiB*	0.00017
Photosystem II 12 kDa extrinsic protein	*psbU*	0.00613
Slr0042	*slr0042*	0.01004
Sll1271	*sll1271*	0.01076
Iron uptake protein A2	*futA2*	0.01795
Sll1638	*psbQ*	0.01902
Sll1550	*sll1550*	0.03325
Ferrichrome-iron receptor	*fhuA*	0.03342

The expression of five putative porins, namely, Slr1841, Slr1908, Slr0042, Sll1550, and Sll1271, mainly involved in ion transport (Qiu et al., 2018), showed significant copper-iron proteomic associations (*P* < 0.05) (Table 2). Among them, Slr1841, Slr1908, and Slr0042 were significantly down-regulated under copper as well as iron deduction conditions (Supplementary Table 2). However, Sll1550 was only significantly down-regulated under copper deduction (Cu-deduction group: 0.75-fold; Cu, Fe-dual deduction group: 0.68-fold), whereas Sll1271 was not significantly down-regulated under copper deduction and iron deduction conditions (Supplementary Table 2).

The expression of iron uptake-related proteins, ferrichrome-iron receptor (FhuA) and iron uptake protein A2 (FutA2) showed significant Cu–Fe proteomic association (*P* < 0.05) (Table 2). FhuA is a TonB-dependent OM transporter for iron that can incorporate iron from the extracellular environment and transport them to the periplasmic space (PP). The expression of FhuA in the was significantly up-regulated by 5.86-fold, 1.9-fold, and 6.8-fold in the Fe-deduction group, Cu-deduction group, and Cu, Fe-dual deduction group, respectively (Supplementary Table 2). FutA2 is a periplasmic water-soluble iron importer, which was significantly up-regulated under iron deduction or copper deduction conditions (3.81-fold up-regulation upon Fe deduction, 1.69-fold upon Cu deduction, and 4.18-fold upon Cu, Fe-dual deduction; Supplementary Table 2).

In addition, the expression of proteins related to photosynthetic electron transport, namely, PsbU (Photosystem II 12 kDa extrinsic protein), PsbQ, and Flavodoxin, also showed copper-iron correlation (*P* < 0.05) (Table 2). Among them, only PsbU was differentially expressed under iron deduction (Fe-deduction group: 0.53-fold; Cu–Fe dual deduction group: 0.4-fold), while no significant differential expression was observed under copper deduction (Supplementary Table 1). PsbQ was significantly down-regulated under these three deduction conditions; it was down-regulated by 0.41-fold in the Fe-deduction group, by 0.81-fold in the Cu-deduction group, and by 0.71-fold in the Cu-Fe dual deduction group (Supplementary Table 1). The expression of the electron carrier, Flavodoxin, was significantly up-regulated in response to copper deduction or iron deduction (Fe-deduction group: 4.29-fold; Cu-deduction group: 2.14-fold; Cu, Fe-dual deduction group: 7.51-fold) (*P* < 0.05) (Supplementary Table 2).

## Discussion

### Growth, Photosynthesis, and Cu–Fe Association

The copper or iron deduction affects growth of *Synechocystis* 6803. As shown in Figure 1, iron played a crucial role in the growth of *Synechocystis* 6803, which has been confirmed previously (Odom et al., 1993). This might be due to iron which is an important cofactor in photosynthesis, such as cytochrome *c6* in the electron transport chain and Fe/S cluster proteins in PSI (Geider and La Roche, 1994). Therefore, severe iron deficiency affected photosynthesis. Copper also plays an important role in the growth of cyanobacteria, and studies have found that severe copper deficiency affects the growth of cyanobacteria (Manahan and Smith, 1973; Sandmann and Böger, 1980). In this study, strict copper starvation was not pursued and different levels of copper deduction were used to testify the effect of small-amount changes of copper on *Synechocystis* physiology. Result indicated that the effect was weak in the presence of increased iron. One of the most remarkable roles of copper affecting on cyanobacterial growth is as the binding metal element of plastocyanin participating in electron transfer. When copper is deficient, the iron-binding protein cytochrome *c6* can be functionally replaced with plastocyanin, which alleviates the impact of copper deprivation on cells (Peers and Price, 2006).

In the measurement of the growth curve, the most important finding is that the growth of Synechocystis 6803 shows Cu-Fe association, for example, among F4 condition (iron concentration: 18.75 μM), cells have the best growth rate among C4 condition (copper concentration: 337.5 nM); while among F5 condition (iron concentration: 25 μM), cells have the best growth rate among C3 condition (copper concentration: 225 nM) (Figure 1). The effect of copper and iron on cyanobacterial growth is consistent with previous reports (Sandmann and Malkin, 1983; Ivanov et al., 2000).

The copper or iron deduction affects the photosynthesis of *Synechocystis* 6803. Y(II) reflects the actual photosynthetic efficiency of PSII, and rETR reflects the relative electron transfer rate of PSII, both of which are related to the activity of linear electron transfer (Genty et al., 1989; Krause and Weis, 1991; Maxwell and Johnson, 2000), thereby showing the Cu–Fe reciprocal effect. For example, when the copper concentration was increased from C3 to C4, the optimal iron concentration of the two parameters decreased from F5 to F4 (Figures 2B,C). However, the maximum value of *F*_*v*_/*F*_*m*_, reflecting the potential photosynthetic activity of the five copper concentrations, was observed in the F5 series, indicating that *F*_*v*_/*F*_*m*_ of these Synechocystis 6803 is mainly affected by the iron concentration owing to the wide-ranging influence of iron on the photosynthetic system (Figure 2A; Hopkinson et al., 2007). These results are consistent with previous reports (Abdel-Ghany and Pilon, 2008; Fraser et al., 2013; Georg et al., 2017).

The effect of copper and iron deduction on the growth of Synechocystis 6803 will directly affect the biomass accumulation, and the effect on photosynthesis will affect a series of metabolic processes including carbohydrate metabolism, lipid catabolic process and protein metabolism (detailed description in “General proteomic analyses”).

### Electron Transport Chain-Related Proteins and Cu–Fe Proteomic Association

Analyzing the Cu–Fe proteome association will help explain the physiologically Cu–Fe association of *Synechocystis* 6803. The results of proteomic analysis (Table 2) showed that the expression of Flavodoxin, PsbU, and PsbQ, proteins related to the electron transport chain, showed significant Cu–Fe proteomic association. This may be due to the fact that electron transport in the electron transport chain is performed by Plastocyanin and/or Cytochrome *c* which containing essential Cu or Fe element respectively (Wood, 1978; Ullmann and Kostic, 1995; Cao et al., 2020), hence, some related proteins and physiological reactions (detailed discussion in section “Growth, photosynthesis, and Cu-Fe association”), such as PsbU and PsbQ, showed Cu–Fe proteome association. PsbU and PsbQ, located upstream of the electron transport chain, can protect the electron-donating Mn_4_CaO_5_ cluster (Mummadisetti et al., 2014; Kim and Debus, 2019). Flavodoxins, as electron transfer proteins located downstream of the electron transport chain (Sancho, 2006), can be induced by iron or copper deduction and also showed a significant Cu–Fe proteomic association (Table 1; Inda and Peleato, 2002). This may be a feedback regulation of cells to metal deduction.

The influence of copper or iron deduction on the electron transport chain will further affect the expression of a series of proteins involved in photosynthetic electron transport. On one hand, all proteins related to the photosynthetic system show differential expression, including various subunits of PSII, PSI, and the Cytochrome *b_6_f* complex (Supplementary Table 1). On the other hand, changes in PSII leads to down-regulation of phycobilisome-related protein expression (Supplementary Table 1). These changes are consistent with previous reports (Allen et al., 2008; Rochaix, 2011; Georg et al., 2017; Angeleri et al., 2019). In addition, the influence of copper or iron limitation on electron transfer will affect the formation of electrochemical gradients of protons inside and outside the TM, which in turn affects the synthesis of ATP by ATP synthase (Ferguson and Sorgato, 1982; Van Walraven et al., 1985; Narayan et al., 2011). The lack of ATP synthesis will trigger a series of differential expression of ATP-dependent proteins and related metabolic changes, including those of ribosomal proteins, various tRNA enzymes, and many other proteins, which are in agreements with the results of protein differential expression identified in this study (Supplementary Table 2) and other reports (Allen et al., 2008; Georg et al., 2017; Angeleri et al., 2019).

### Copper and Iron Uptake System and Cu–Fe Proteomic Association

Seven proteins related to copper and iron uptake, distributed in OM, PP, and TM, also showed significant Cu–Fe proteomic association (Table 2), except for the three TM proteins in the electron transport chain. Therefore, significant Cu–Fe proteomic association may also exist during the uptake of copper and iron from OM to PP and that this association was maintained during the transport of copper and iron through IM and TM.

Copper and iron were supposed mainly carried out from OM to PP in two ways. On the one hand, the OM porins of cyanobacteria promote the passive uptake of iron (Nikaido, 2003; Qiu et al., 2018) and may also be related to copper transfer (Speer et al., 2013; Stewart et al., 2019). On the other hand, copper and iron can also be actively transported to the PP via some transporters on the OM, including the TonB-dependence transporter (TBDT) and ABC transporter (Schöffler and Braum, 1989; Köster, 2001; Yue et al., 2003).

We observed that porins Slr1841, Slr1908, and Slr0042 were significantly down-regulated under copper deduction and iron deduction conditions; Sll1550 was only significantly down-regulated under copper deduction conditions, while Sll1271 was not significantly down-regulated under copper deduction and iron deduction conditions (Supplementary Table 2). This is consistent with previous results regarding copper- or iron-related stress (Kowata et al., 2017; Angeleri et al., 2019; Kojima and Okumura, 2020). Studies have shown that the deletion of Sll0772, Sll1271, Sll1550, and Slr0042 retarded the growth of Synechocystis 6803 in iron-deficient media (Qiu et al., 2018), suggesting that these porins are associated with iron uptake. Based on this and combined with the Cu–Fe proteomic association results of this study (Table 1), we speculated that the porins, Slr1841, Slr1908, and Slr0042, may be involved in the uptake of copper and iron from the extracellular environment and their transport to the periplasm, while Sll1550 is only related to copper uptake. Reports show that expression of Sll1550 is induced in the knock outs of its homologs, Slr1908 or Slr1841 (Kowata et al., 2017; Kojima and Okumura, 2020). This suggests that these porins coordinate with each other during copper or iron uptake and possibly maintain the normal transport of iron or the dynamic balance between copper and iron uptake. A significant Cu–Fe reciprocal effect on the expression of Sll1271 was observed (Table 1), although it was not significantly down-regulated under copper or iron deduction conditions. Currently, reports regarding the function of Sll1271 are limited.

In addition to porins, iron or copper can also be actively transported to the periplasm via certain transporters. At present, reports regarding copper OM transfer proteins are limited; however, several iron OM transporters have been discovered, such as iron ABC transporter-FutA1, TonB-dependence transporter-FhuA1, and iron uptake transporter-IutA (Hantke and Braun, 1975; Moeck et al., 1997; Ferguson et al., 1998; Pawelek et al., 2006). Although whether FhuA can directly transport copper is not known, we observed that FhuA1 can respond to copper deduction (up 1.9-fold) or iron deduction (up 5.86-fold) via differential expression (Supplementary Table 2) and significantly responds to the Cu–Fe reciprocal effect (Table 1). Similarly, the TonB-dependent iron transporter, -IacT, in *Anabaena* can also be significantly up-regulated in response to copper deduction (Nicolaisen et al., 2010). We speculated that the up-regulation of these two TonB-dependent iron transporters under copper deduction conditions may be related to TonB (Nicolaisen et al., 2010), as well as other related periplasmic proteins.

Recent studies have shown that after copper and iron are incorporated from the extracellular environment to the periplasmic space, some periplasmic transporters play important roles in maintaining the balance of copper and iron uptake. For example, the periplasmic protein FutA2 can not only participate in the uptake of Fe^3+^, but also plays a vital role in copper uptake (Badarau et al., 2008). Absence of FutA2 will lead to insufficient copper supply in the thylakoid cavity, as FutA2 can restrict the abnormal binding of Fe^3+^ to important binding sites of copper by isolating Fe^3+^, thereby indirectly assisting in passage of copper from the periplasm through the inner plasma membrane (IM) and TM to the thylakoid cavity. This explains our observation that FutA2 can be significantly up-regulated in response to copper or iron deduction and shows a Cu-Fe proteomic association (Table 1 and Supplementary Table 2), which is reflected in the connection between the TM and OM-PP-IM.

In summary, these Cu–Fe associated proteins play important role in the process of copper and iron uptake from OM to PP and then through IM and TM. Previous studies have only reported Cu–Fe association of individual proteins, and have not been linked to other Cu–Fe associated uptake proteins (Hantke and Braun, 1975; Moeck et al., 1997; Ferguson et al., 1998; Pawelek et al., 2006; Badarau et al., 2008; Speer et al., 2013; Stewart et al., 2019). However, we conducted a systematic analysis of these Cu–Fe associated proteins. And we think that there is a Cu–Fe proteomic association in the process of Synechocystis 6803 uptake of copper and iron from the extracellular environment to the thylakoid cavity via the OM-PP-IM-TM uptake system.

### Cu–Fe Proteomic Association With a Hypothetical Uptake System

**FIGURE 4:**
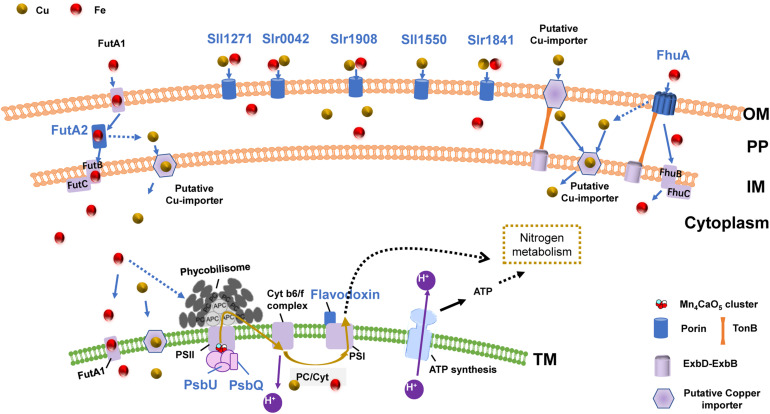
The OM-PP-IM-TM Cu–Fe uptake system and photosynthetic electron transport chain. Proteins shown in blue: the protein expression shows significant Cu–Fe proteomic association; Solid arrow: direct influence; Dashed arrow: indirect influence.

Based on the above results regarding growth, chlorophyll fluorescence, and expression of certain proteins, as well as the important roles of copper and iron in the photosynthetic electron transport chain, we proposed a hypothesis (Figure 4): during copper or iron deduction, Synechocystis 6803 responds to and regulates intracellular copper and iron content via an OM-PP-IM-TM Cu-Fe uptake system to maintain copper and iron homeostasis. The response of this Cu–Fe uptake system continues to affect the photosynthetic electron transport chain, which ultimately affects photosynthesis and inhibits cell growth. This feedback regulation mechanism involves association within the Cu–Fe proteome, which is mainly affected by the Cu–Fe supply of the electron transport chain.

The proposed role of the OM-PP-IM-TM uptake system is supported by numerous evidences regarding membrane connectivity. For example, structural biology evidences have identified a thylakoid convergence region inside the cytoplasmic membrane, which proves that there may be a certain degree of membrane structural continuity between the thylakoid cavity, periplasmic and extracellular environment (Liu and Zhang, 2019; Rast et al., 2019). Studies have also shown that the absence of OM porins such as Slr0042, Slr1841, and Slr1908 induces the release of functional proteins in the thylakoid cavity, including Plastocyanin, Cytochrome *c*, PsbU, and other photosynthetic electron transport-related proteins (Kojima and Okumura, 2020). This may be related to the connectivity of the membrane system, which will affect the electron transport chain.

The connectivity of the OM-PP-IM-TM membrane system suggests that the copper–iron transport is not limited to a single protein, but is possibly a consequence of the coordination of the entire copper–iron uptake system and the photosynthetic electron transport chain; for example, the synergy between active absorption and diffusion absorption in the OM (Qiu et al., 2018), and the functions of periplasmic proteins during the transfer of copper and iron from the periplasm into the membrane might be coordinated.

However, only few reports on copper uptake and copper-iron homeostasis are currently available, hence, the following questions have not been resolved: (1) what is the specific mechanism underlying the differential expression of several porins in response to copper and iron deduction? (2) can porins directly transport copper or iron? (3) how does the iron TonB-dependent transporter affect copper uptake? We look forward to verification of the hypothesis to address these questions, which needs a lot of future work.

## Conclusion

Our research shows that the growth of Synechocystis 6803 was restricted during copper or iron deduction and was exhibited a Cu–Fe reciprocal effect under different copper and iron culture concentrations. Similarly, the data of chlorophyll fluorescence parameter Y(II) and rETR related to linear electron transfer activity also showed a Cu–Fe reciprocal effect. The proteomics results revealed that the three proteins (Flavodoxin, PsbU, and PsbQ) related to the electron transport chain showed significant Cu–Fe proteomic association. And many proteins involved in photosynthetic electron transport are also significantly differentially expressed, which will affect the photosynthetic metabolism and in turn affect other energy metabolism, such as carbohydrate metabolism, lipid catabolic process, and protein metabolism. In addition, the Cu–Fe proteomic association of Synechocystis 6803 is also manifested in the process of copper and iron uptake, which involves five porins (Slr1841, Slr1908, Slr0042, Sll1550, and Sll1271) and two iron transporters (FhuA and FutA2).

The above results reveal that in order to meet the intracellular requirements of copper or iron under deduction conditions, especially for the electron transport chain, these metals can be transferred into the cell via the uptake system (the OM-PP-IM-TM uptake system). Due to the functional substitution of copper-binding protein and iron-binding protein in the electron transport chain, some proteins involved in electron transport showed significant Cu-Fe proteomic association. In addition, the effect of copper and/or iron deduction on the photosynthetic electron transport chain will further affect the growth of *Synechocystis* 6803, and show a significant Cu-Fe association. Our research provides a theoretical basis for the regulatory link between copper and iron homeostasis in cyanobacteria, which will clarify the role of these two metal elements in cyanobacterial energy metabolism and biomass accumulation.

## Data Availability Statement

The data presented in the study are deposited in the ProteomeXchange repository, accession number PXD024873.

## Author Contributions

Y-CW and SQ had completed the experimental design and financial support. Q-MR, YW, and Y-YM had completed the data analysis. Z-HZ had completed the all experiments and thesis writing. All the authors read and approved the final manuscript.

## Conflict of Interest

The authors declare that the research was conducted in the absence of any commercial or financial relationships that could be construed as a potential conflict of interest.
