# Supplementary material for: Reciprocal Effect of Copper and Iron Regulation on the Proteome of Synechocystis sp. PCC 6803
Source: Front Bioeng Biotechnol. 2021 May 10;9:673402. doi: 10.3389/fbioe.2021.673402 (PMC8141849; doi:10.3389/fbioe.2021.673402)
Supplement: Supplementary file 12 [file Data_Sheet_12.docx]

Supplementary Material

# Supplementary Figures





**Supplementary Figure 1.** Figure S1 Standard curve of optical density at 730nm of *Synechocystis* sp. PCC6803 showing the corresponding optical density value measured with normal spectrophotometer with standard glass cuvette cell (1.0 cm path length) and the microplate reader (Shanpu SuPerMax 3100, Shanghai, China) used in this study.

# Supplementary Tables

**Supplementary Table 1.** Changes in expression of photosynthesis- and light-harvesting system- related proteins under Cu or/and Fe deduction

| **Protein** | **Gene** | **Fe-deduction** | **Cu-deduction** | **Cu,Fe-dual deduction** | **Description** |
| --- | --- | --- | --- | --- | --- |
| **Photosystem II** |  |  |  |  |  |
| Photosystem II protein D1 2 | *psbA2* | 0.66^#^ | 0.64^#^ | 0.79^#^ | Photosystem II protein D1 2 targeted to photodamage and rapid turnover |
| Photosystem II lipoprotein Psb27 | *psb27* | 0.62 | 0.83 | 0.84^#^ | Photosystem II lipoprotein Psb27 |
| Photosystem II reaction center Psb28 protein | *psb28* | 1.54 | 0.95^#^ | 2.51 | Photosystem II reaction center Psb28 protein |
| Photosystem II reaction center Psb28 protein | *slr1739* | 0.68 | 0.81^#^ | 1.11^#^ | Photosystem II reaction center Psb28 protein |
| Photosystem II CP47 reaction center protein | *psbB* | 0.48 | 0.47 | 0.47 | Photosystem II CP47 reaction center protein |
| Photosystem II CP43 reaction center protein | *psbC* | 0.59 | 0.63^#^ | 0.70^#^ | Photosystem II CP43 reaction center protein |
| Photosystem II D2 protein | *psbD* | 0.54 | 0.54 | 0.69^#^ | Photosystem II D2 protein |
| Cytochrome b559 subunit alpha | *psbE* | 0.27 | 0.91^#^ | 0.30 | Cytochrome b559 subunit alpha |
| Cytochrome b559 subunit beta | *psbF* | 0.27 | 0.59 | 0.31 | Cytochrome b559 subunit beta |
| Photosystem II reaction center protein H | *psbH* | 0.29 | 0.61 | 0.22 | Photosystem II reaction center protein H |
| Photosystem II reaction center protein L | *psbL* | 0.29 | 1.14^#^ | 0.18 | Photosystem II reaction center protein L |
| Photosystem II manganese-stabilizing polypeptide | *psbO* | 0.41 | 0.81 | 0.71 | Photosystem II manganese-stabilizing polypeptide |
| Photosystem II 12 kDa extrinsic protein | *psbU* | 0.53 | 0.96^#^ | 0.40 | Photosystem II 12 kDa extrinsic protein associated with stabilizing the oxygen evolving machinery of photosynthesis |
| Cytochrome c-550 | *psbV* | 0.33 | 0.95^#^ | 0.30 | Cytochrome c-550 |
| Photosystem II protein Y | *psbY* | 0.28 | 0.90^#^ | 0.19 | Photosystem II protein Y |
| Slr0147 protein | *slr0147* | 0.07 | 0.47 | 0.21 | Component of the PSII assembly protein operon |
| Slr0149 protein | *slr0149* | 0.09 | 0.59 | 0.14 | Component of the PSII assembly protein operon |
| Slr0151 protein | *slr0151* | 0.25 | 0.63 | 0.49 | Component of the PSII assembly protein operon |
| **Photosystem I** |  |  |  |  |  |
| Photosystem I P700 chlorophyll a apoprotein A1 | *psaA* | 0.27 | 0.56 | 0.29 | Photosystem I P700 chlorophyll a apoprotein A1 |
| Photosystem I P700 chlorophyll a apoprotein A2 | *psaB* | 0.22 | 0.5 | 0.21 | Photosystem I P700 chlorophyll a apoprotein A2 |
| Photosystem I iron-sulfur center | *psaC* | 0.42 | 0.67 | 0.52 | Photosystem I iron-sulfur center |
| Photosystem I reaction center subunit II | *psaD* | 0.11 | 0.74 | 0.14 | Photosystem I reaction center subunit II |
| Photosystem I reaction center subunit IV | *psaE* | 0.23 | 0.43 | 0.39 | Photosystem I reaction center subunit IV |
| Photosystem I reaction center subunit III | *psaF* | 0.09 | 0.8 | 0.14 | Photosystem I reaction center subunit III |
| Photosystem I reaction center subunit IX | *psaJ* | 0.59 | 0.79^#^ | 0.62 | Photosystem I reaction center subunit IX |
| Photosystem I reaction center subunit PsaK 2 | *psaK2* | 0.15 | 0.75 | 0.10 | Photosystem I reaction center subunit PsaK 2 |
| Photosystem I reaction center subunit XI | *psaL* | 0.18 | 0.60 | 0.32 | Photosystem I reaction center subunit XI |
| Photosystem I reaction center subunit XII | *psaM* | 0.14 | 0.84^#^ | 0.10 | Photosystem I reaction center subunit XII |
| **Cytochrome b6/f complex** |  |  |  |  |  |
| Cytochrome b6 | *petB* | 0.29 | 0.59 | 0.38 | Cytochrome b6 |
| Cytochrome f | *petA* | 0.28 | 0.7 | 0.90^#^ | Cytochrome f |
| Cytochrome b6/f complex iron-sulfur subunit | *petC* | 0.66^#^ | 0.80^#^ | 0.12 | Cytochrome b6/f complex iron-sulfur subunit |
| Cytochrome b6-f complex subunit 4 | *petD* | 0.11 | 0.64 | 0.17 | Cytochrome b6-f complex subunit 4 |
| Cytochrome b6-f complex subunit 5 | *petG* | 0.31 | 1.04^#^ | 0.26 | Cytochrome b6-f complex subunit 5 |
| **Photosynthesis electron transport** | |  |  |  |  |
| Plastocyanin | *PetE* | 1.44 | 0.33 | 0.52 | Plastocyanin |
| Ferredoxin-1 | *PetF* | 0.30 | 0.60 | 0.26 | Ferredoxin-1 |
| Ferredoxin--NADP reductase | *PetH* | 0.65 | 0.86^#^ | 0.78 | Ferredoxin--NADP reductase |
| Ferredoxin-thioredoxin reductase, catalytic chain | ftrC | 0.32 | 0.94^#^ | 0.35 | Ferredoxin-thioredoxin reductase, catalytic chain |
| Ferredoxin-thioredoxin reductase, variable chain | ftrV | 2.12^#^ | 2.07^#^ | 5.24 | Ferredoxin-thioredoxin reductase, variab chain |
| Cytochrome c6 | *PetJ* | 0.77^#^ | 18.06 | 1.04^#^ | Cytochrome c553 |
| **Light-harvesting antenna** |  |  |  |  |  |
| Allophycocyanin alpha chain | *apcA* | 0.40 | 0.60 | 0.75 | Allophycocyanin alpha chain |
| Allophycocyanin beta chain | *apcB* | 0.40 | 0.56 | 0.65 | Allophycocyanin beta chain |
| Phycobilisome 7.8 kDa linker polypeptide, L_C_ | *apcC* | 0.31 | 0.75 | 0.20 | Phycobilisome 7.8 kDa linker polypeptide, allophycocyanin-associated, core LC |
| Allophycocyanin subunit alpha-B | *apcD* | 0.46 | 0.72 | 0.83 | Allophycocyanin subunit alpha-B |
| Phycobiliprotein ApcE | *apcE* | 0.66 | 0.77^#^ | 0.92^#^ | Phycobiliprotein ApcE |
| Allophycocyanin subunit beta-18 | *apcF* | 0.56 | 0.75 | 0.96^#^ | Allophycocyanin subunit beta-18 |
| C-phycocyanin alpha chain | *cpcA* | 0.38 | 0.72 | 0.66 | C-phycocyanin alpha chain |
| C-phycocyanin beta chain | *cpcB* | 0.31 | 0.55 | 0.61 | C-phycocyanin beta chain |
| Phycobilisome rod-core linker polypeptide CpcG | *cpcG* | 0.24 | 0.77^#^ | 0.56 | Phycobilisome rod-core linker polypeptide CpcG LRC |
| Phycobilisome rod-core linker polypeptide CpcG | *cpcG* | 0.38 | 0.84^#^ | 0.58 | Phycobilisome rod-core linker polypeptide CpcG LRC |
| Phycobilisome 32.1 kDa linker polypeptide, LR 1 | *cpcC1* | 0.24 | 0.68 | 0.59 | Phycobilisome 32.1 kDa linker polypeptide, phycocyanin-associated, rod 1 LR1 |
| Phycobilisome 32.1 kDa linker polypeptide, LR 2 | *cpcC2* | 0.39 | 0.85^#^ | 1.02^#^ | Phycobilisome 32.1 kDa linker polypeptide, phycocyanin-associated, rod 2 LR2 |
| Phycobilisome 8.9 kDa linker polypeptide, LR | *cpcD* | 0.57 | 0.78 | 0.63 | Phycobilisome 8.9 kDa linker polypeptide, phycocyanin-associated, rod LR |
| Phycocyanobilin lyase subunit alpha | *cpcE* | 0.67 | 1.08^#^ | 1.31^#^ | Phycocyanobilin lyase subunit alpha |
| Phycocyanin alpha phycocyanobilin lyase CpcF | *cpcF* | 0.72 | 1.02^#^ | 1.12^#^ | Phycocyanin alpha phycocyanobilin lyase CpcF |
| Iron stress-induced chlorophyll-binding protein | *isiA* | 4.41 | 1.50^#^ | 4.52 | Iron stress-induced chlorophyll-binding protein |
| **NADPH** |  |  |  |  |  |
| Uncharacterized SufE-like protein slr1419 | *slr1419* | 1.79 | 1.11^#^ | 2.65 | Photosynthesis |
| NAD(P)H-quinone oxidoreductase subunit J | *ndhJ* | 1.13^#^ | 0.85^#^ | 2.13 | Oxidative phosphorylation |
| Ketol-acid reductoisomerase (NADP(+)) | *ilvC* | 1.10^#^ | 1.07^#^ | 1.81 | Valine, Leucine and isoleucine biosynthesis; Pantothenate and CoA biosynthesis |
| Enoyl-[acyl-carrier-protein] reductase [NADH] FabI | *fabI* | 1.14^#^ | 1.37^#^ | 1.72 | Fatty acid biosynthesis  Biotin metabolism |
| NAD(P)H-quinone oxidoreductase subunit M | *ndhM* | 1.34 | 0.98^#^ | 1.62 | Oxidative phosphorylation |
| Isocitrate dehydrogenase [NADP] | *icd* | 1.06^#^ | 0.92^#^ | 1.50 | Glutathione metabolism;  Carbon fixation pathways in prokaryotes;  Citrate cycle (TCA cycle); Peroxisome |
| NAD(P)H-quinone oxidoreductase subunit K 1 | *ndhK1* | 1.19^#^ | 1.14^#^ | 1.45 | Oxidative phosphorylation |
| NAD(P)H-quinone oxidoreductase subunit H | *ndhH* | 1.33^#^ | 0.91^#^ | 1.41 | Oxidative phosphorylation |
| Ferredoxin--NADP reductase | *petH* | 0.65 | 0.86^#^ | 0.78 | Photosynthesis |
| NAD(P)H-quinone oxidoreductase chain 5 | *ndhF* | 0.67 | 0.79^#^ | 0.69^#^ | Oxidative phosphorylation |
| NAD(P)H-quinone oxidoreductase subunit O | *ndhO* | 0.87^#^ | 0.77^#^ | 0.56 | Quinone subunit |
| NAD(P)H-quinone oxidoreductase chain 6 | *ndhG* | 0.93^#^ | 1.35 | 0.54 | Oxidative phosphorylation |
| NAD(P)H-quinone oxidoreductase subunit 4L | *ndhE* | 0.48^#^ | 0.75^#^ | 0.30 | Oxidative phosphorylation |

^#^: *P* > 0.05

| **Supplementary Table 2.** Changes in expression of non-photosynthetic-related proteins under Cu or/and Fe deduction | | | | | |
| --- | --- | --- | --- | --- | --- |
| **Protein** | **Gene** | **Fe-deduction** | **Cu-deduction** | **Cu, Fe-dual deduction** | **Description** |
| **Iron-related proteins** |  |  |  |  |  |
| Flavodoxin | *isiB* | 4.29 | 2.14 | 7.51 | Electron-transfer proteins |
| Ferrichrome-iron receptor | *fhuA* | 5.86 | 1.90 | 6.80 | Ferric receptor |
| Iron uptake protein A1 | *futA1* | 3.49 | 1.50 | 4.54 | ABC transporters |
| Iron uptake protein A2 | *futA2* | 3.81 | 1.69 | 4.18 | ABC transporters |
| Ssl2245 protein | *ssl2245* | 2.99 | 1.03^#^ | 3.65 | Toxin-Antitoxin system |
| Ferric aerobactin receptor | *iutA* | 4.59 | 1.67^#^ | 3.83 | Ferric receptor |
| Putative sulfur carrier protein slr0821 | *slr0821* | 2.35 | 1.17^#^ | 1.89 | Sulfur relay system |
| Sll1130 protein | *sll1130* | 1.36 | 1.35 | 0.96^#^ | Toxin-Antitoxin system |
| Dihydroxy-acid dehydratase | *ilvD* | 0.76 | 0.92^#^ | 0.79^#^ | Valine, Leucine and isoleucine biosynthesis; Pantothenate and CoA biosynthesis |
| Superoxide dismutase [Fe] | *sodB* | 0.47 | 0.69 | 0.71^#^ | Longevity regulating pathway; MAPK (mitogen-activated protein kinase) signaling pathway; Peroxisome |
| Ferredoxin | *slr0148* | 0.36^#^ | 0.58^#^ | 0.47^#^ | Ferredoxin |
| Group 1 truncated hemoglobin GlbN | *glbN* | 0.48 | 0.83 | 0.33 | Heme-containing globular proteins |
| Iron-regulated protein | *frpC* | 0.48 | 1.25 | 0.18 | Iron-regulated protein |
| Ferredoxin component | *slr1205* | 0.20 | 0.95^#^ | 0.10 | Ferredoxin component |
| Aconitate hydratase B | *acnB* | 0.08 | 1.59 | 0.08 | Propanoate metabolism; Carbon fixation pathways in prokaryotes; Glyoxylate and dicarboxylate metabolism; Citrate cycle (TCA cycle) |
| **Carbon / Nitrogen metabolism** |  |  |  |  |  |
| Glutamate--ammonia ligase | *glnN* | 0.60^#^ | 1.10^#^ | 3.63 | Nitrogen metabolism |
| DNA-directed RNA polymerase subunit alpha | *rpoA* | 1.69 | 1.32 | 3.20 | RNA polymerase |
| Hydroperoxy fatty acid reductase gpx1 | *gpx1* | 2.29 | 1.30 | 3.10 | Hydroperoxy fatty acid reductase |
| 6-phosphogluconate dehydrogenase, decarboxylating | *gnd* | 2.42 | 1.22^#^ | 2.61 | Glutathione metabolism;  Pentose phosphate pathway |
| Glutamine synthetase | *glnA* | 1.14^#^ | 1.24 | 2.33 | Nitrogen metabolism |
| Biotin carboxyl carrier protein of acetyl-CoA carboxylase | *accB* | 1.39^#^ | 1.05^#^ | 2.27 | Carbon fixation pathways in prokaryotes;  Fatty acid biosynthesis |
| Glucose-6-phosphate isomerase | *pgi* | 1.29 | 1.08^#^ | 2.09 | Starch and sucrose metabolism;  Pentose phosphate pathway;  Glycolysis |
| Probable phosphoketolase | *slr0453* | 1.94 | 1.18^#^ | 2.05 | Carbon fixation in photosynthetic organisms |
| Alpha-1,4 glucan phosphorylase | *glgP* | 1.83 | 1.32 | 2.02 | Necroptosis;  Insulin signaling pathway;  Insulin resistance;  Glucagon signaling pathway;  Biofilm formation;  Starch and sucrose metabolism |
| Ribulose bisphosphate carboxylase large chain | *cbbL* | 1.34^#^ | 1.12^#^ | 1.73 | Carbon fixation in photosynthetic organisms |
| Enoyl-[acyl-carrier-protein] reductase [NADH] FabI | *fabI* | 1.14^#^ | 1.37^#^ | 1.72 | Fatty acid biosynthesis |
| 3-hydroxyacyl-[acyl-carrier-protein] dehydratase FabZ | *fabZ* | 1.00^#^ | 1.07^#^ | 1.70 | Fatty acid biosynthesis |
| Phosphoglucomutase | *pgm* | 1.16^#^ | 1.08^#^ | 1.61 | Starch and sucrose metabolism;  Pentose phosphate pathway;  Glycolysis |
| Aminopeptidase | *ape2* | 2.09 | 1.69 | 1.60 | Glutathione metabolism |
| Phosphoribulokinase | *prk* | 1.16^#^ | 0.92^#^ | 1.55 | Carbon fixation in photosynthetic organisms |
| Enolase | *eno* | 0.94^#^ | 1.04^#^ | 1.53 | Glycolysis |
| 3-oxoacyl-[acyl-carrier-protein] reductase | *fabG* | 1.06^#^ | 0.97^#^ | 1.51 | Fatty acid biosynthesis |
| Isocitrate dehydrogenase [NADP] | *icd* | 1.06^#^ | 0.92^#^ | 1.50 | Carbon fixation pathways in prokaryotes;  Citrate cycle (TCA cycle) |
| Cyanate hydratase | *cynS* | 0.62 | 0.83 | 1.49 | Nitrogen metabolism |
| Carbonic anhydrase | *icfA* | 2.09 | 1.08^#^ | 1.43^#^ | Nitrogen metabolism |
| Glucose-6-phosphate 1-dehydrogenase | *zwf* | 1.73 | 1.31 | 1.38^#^ | Glutathione metabolism; Pentose phosphate pathway |
| Pyruvate dehydrogenase E1 component subunit alpha | *pdhA* | 0.86^#^ | 0.81^#^ | 1.32 | Glycolysis; Citrate cycle (TCA cycle) |
| Pyruvate kinase 2 | *pyk2* | 1.62 | 1.11^#^ | 1.30^#^ | Glycolysis |
| Triosephosphate isomerase | *tpiA* | 1.18^#^ | 1.06^#^ | 1.27 | Glycolysis;  Carbon fixation in photosynthetic organisms |
| Ribose-5-phosphate isomerase A | *rpiA* | 0.89^#^ | 0.94^#^ | 1.24 | Carbon fixation in photosynthetic organisms;  Pentose phosphate pathway |
| Glutathione S-transferase | *gst* | 1.10^#^ | 0.62 | 1.13^#^ | Glutathione metabolism |
| DNA topoisomerase 1 | *topA* | 2.42 | 1.52 | 0.78^#^ | DNA topoisomerase |
| Biotin carboxylase | *accC* | 1.73 | 2.07 | 0.76^#^ | Propanoate metabolism;  Carbon fixation pathways in prokaryotes;  Fatty acid biosynthesis;  Pyruvate metabolism |
| Fructose-bisphosphate aldolase class 1 | *fda* | 0.74 | 0.79^#^ | 0.76 | Carbon fixation in photosynthetic organisms; Glycolysis; Fructose and mannose metabolism;  Pentose phosphate pathway |
| Fructose-bisphosphate aldolase class 2 | *fbaA* | 0.36 | 0.70 | 0.75 | Carbon fixation in photosynthetic organisms; Fructose and mannose metabolism; Glycolysis;  Pentose phosphate pathway |
| Acetyl-coenzyme A carboxylase carboxyl transferase subunit alpha | *accA* | 0.61 | 0.84^#^ | 0.73 | Carbon fixation pathways in prokaryotes;  Fatty acid biosynthesis |
| Nitrate reductase | *narB* | 1.97 | 2.01 | 0.67^#^ | Nitrogen metabolism |
| Ferredoxin--nitrite reductase | *nirA* | 0.45 | 0.84^#^ | 0.67 | Nitrogen metabolism |
| Phosphomannose isomerase | *rfbM* | 0.78 | 0.84^#^ | 0.67 | Fructose and mannose metabolism |
| Ribulose bisphosphate carboxylase small chain | *cbbS* | 0.50 | 0.59 | 0.59 | Carbon fixation in photosynthetic organisms |
| UvrABC system protein A | *uvrA* | 1.35^#^ | 2.21 | 0.55^#^ | Nucleotide excision repair |
| DNA topoisomerase 4 subunit A | *parC* | 1.86 | 1.64 | 0.40 | DNA topoisomerase |
| Glycine--tRNA ligase alpha subunit | *glyQ* | 1.48^#^ | 2.56^#^ | 0.37 | Aminoacyl-tRNA biosynthesis |
| Ferredoxin-dependent glutamate synthase 1 | *gltB* | 0.45 | 0.90^#^ | 0.34 | Nitrogen metabolism |
| ATP-dependent Clp protease ATP-binding subunit ClpX | *clpX* | 1.65 | 1.73 | 0.32 | Cell cycle |
| ATP-dependent 6-phosphofructokinase 1 | *pfkA1* | 0.96^#^ | 2.27 | 0.30 | Glycolysis / Gluconeogenesis;  Pentose phosphate pathway;  Methane metabolism;  Fructose and mannose metabolism;  Glycolysis Galactose metabolism |
| Ribose phosphate isomerase B | *rpiB* | 0.55 | 1.46 | 0.29 | Fructose and mannose metabolism;  Carbon fixation in photosynthetic organisms; Pentose phosphate pathway |
| Alanine--tRNA ligase | *alaS* | 1.43 | 1.33^#^ | 0.27 | Tryptophan metabolism;  Drug metabolism - other enzymes;  Phenylpropanoid biosynthesis;  Phenylalanine metabolism |
| Nitrate transport ATP-binding protein NrtC | *nrtC* | 0.98^#^ | 0.85^#^ | 0.26 | Nitrogen metabolism |
| Aconitate hydratase B | *acnB* | 0.08 | 1.59 | 0.08 | Glyoxylate and dicarboxylate metabolism;  Carbon fixation pathways in prokaryotes;  Citrate cycle (TCA cycle); Propanoate metabolism |
| **Ribosome** |  |  |  |  |  |
| 50S ribosomal protein L31 | *rpmE* | 1.32^#^ | 1.12^#^ | 3.9 | Ribosome |
| 30S ribosomal protein S19 | *rpsS* | 2.43 | 1.68 | 3.61 | Ribosome |
| 50S ribosomal protein L33 | *rpmG* | 1.89 | 1.51^#^ | 1.95 | Ribosome |
| 50S ribosomal protein L5 | *rplE* | 0.74 | 0.91^#^ | 1.57 | Ribosome |
| 50S ribosomal protein L10 | *rplJ* | 0.64 | 0.90^#^ | 1.52 | Ribosome |
| 50S ribosomal protein L6 | *rplF* | 0.63 | 0.97^#^ | 1.5 | Ribosome |
| 50S ribosomal protein L11 | *rplK* | 0.74 | 0.88^#^ | 1.37 | Ribosome |
| 30S ribosomal protein S2 | *rpsB* | 0.58 | 0.83 | 1.32 | Ribosome |
| 50S ribosomal protein L4 | *rplD* | 0.65 | 0.94^#^ | 1.06^#^ | Ribosome |
| 30S ribosomal protein S11 | *rpsK* | 0.63 | 0.86 | 1.01 | Ribosome |
| 50S ribosomal protein L16 | *rplP* | 0.78 | 0.80^#^ | 0.91^#^ | Ribosome |
| 50S ribosomal protein L3 | *rplC* | 0.65 | 0.87^#^ | 0.88^#^ | Ribosome |
| 50S ribosomal protein L13 | *rplM* | 0.67 | 0.90^#^ | 0.87^#^ | Ribosome |
| 50S ribosomal protein L17 | *rplQ* | 0.64 | 0.95^#^ | 0.69 | Ribosome |
| 30S ribosomal protein S10 | *rpsJ* | 0.62 | 0.92^#^ | 0.62 | Ribosome |
| 30S ribosomal protein S16 | *rpsP* | 0.56 | 0.79 | 0.59 | Ribosome |
| 30S ribosomal protein S18 | *rpsR* | 0.65 | 1.14^#^ | 0.58 | Ribosome |
| 50S ribosomal protein L23 | *rplW* | 0.49 | 0.80 | 0.58 | Ribosome |
| 50S ribosomal protein L25 | *rplY* | 0.51 | 0.78 | 0.58 | Ribosome |
| 30S ribosomal protein S17 | *rpsQ* | 0.65 | 0.81 | 0.57 | Ribosome |
| 50S ribosomal protein L32 | *rpmF* | 0.45 | 0.89^#^ | 0.48 | Ribosome |
| 50S ribosomal protein L27 | *rpmA* | 0.49 | 0.76 | 0.46 | Ribosome |
| 30S ribosomal protein S12 | *rpsL* | 1.02^#^ | 1.36 | 0.24 | Ribosome |
| 50S ribosomal protein L34 | *rpmH* | 0.36 | 0.98^#^ | 0.2 | Ribosome |
|  |  |  |  |  |  |
|  |  |  |  |  |  |
|  |  |  |  |  |  |
|  |  |  |  |  |  |
|  |  |  |  |  |  |
|  |  |  |  |  |  |
|  |  |  |  |  |  |
|  |  |  |  |  |  |
|  |  |  |  |  |  |
|  |  |  |  |  |  |
|  |  |  |  |  |  |
|  |  |  |  |  |  |
|  |  |  |  |  |  |
|  |  |  |  |  |  |
| **ATPsynthase** |  |  |  |  |  |
| ATP synthase subunit beta | *atpD* | 1.03^#^ | 1.17^#^ | 2.3 | Oxidative phosphorylation; Photosynthesis |
| ATP synthase subunit alpha | *atpA* | 1.02^#^ | 1.05^#^ | 1.6 | Oxidative phosphorylation; Photosynthesis |
| ATP synthase subunit delta | *atpH* | 0.66 | 0.95^#^ | 1.3 | Oxidative phosphorylation; Photosynthesis |
| ATP synthase epsilon chain | *atpC* | 0.74 | 0.97^#^ | 1.20^#^ | Oxidative phosphorylation; Photosynthesis |
| ATP synthase gamma chain | *atpG* | 0.63 | 0.88^#^ | 1.11^#^ | Oxidative phosphorylation; Photosynthesis |
| ATP synthase subunit c | *atpE* | 0.62 | 0.99^#^ | 0.61 | Oxidative phosphorylation; Photosynthesis |
| ATP synthase subunit b | *atpF* | 0.41 | 0.74 | 0.39 | Oxidative phosphorylation; Photosynthesis |
| ATP synthase subunit b' | *atpG* | 0.36 | 0.81 | 0.37 | Oxidative phosphorylation; Photosynthesis |
| **Chloroplast** |  |  |  |  |  |
| Ycf23 protein (cyanobacterial ortholog) | *ycf23* | 5.43 | 1.65 | 4.64 | Chloroplast-related protein |
| Ycf35 (cyanobacterial ortholog) | *ycf35* | 2.64 | 1.82 | 4.19 | Chloroplast-related protein |
| Chloroplast import-associated channel IAP75 | *IAP75* | 0.81 | 0.8 | 0.7 | Chloroplast-related protein |
| Chloroplast membrane-associated 30 kD protein | *im30* | 0.44 | 0.75^#^ | 0.37 | Chloroplast-related protein |
| **Membrane** |  |  |  |  |  |
| Membrane-bound lytic transglycosylase A | *mltA* | 0.93^#^ | 1.47 | 1.51 | Membrane-related enzyme |
| Membrane protein | *pilM* | 0.64 | 0.72 | 1.26 | Membrane protein |
| Protein-export membrane protein SecG | *ycf47* | 0.79 | 1.02^#^ | 0.86^#^ | Bacterial secretion system; Protein export; Quorum sensing |
| Membrane-associated protein slr1513 | *slr1513* | 0.49 | 0.81 | 0.66 | Membrane protein |
| **Thylakoid** |  |  |  |  |  |
| Thylakoid membrane protein slr1796 | *slr1796* | 1.33^#^ | 1.40 | 2.55 | Thylakoid membrane protein |
| Putative thylakoid lumen peptidyl-prolyl cis-trans isomerase sll0408 | *sll0408* | 1.97 | 1.38 | 2.07 | Thylakoid lumen protein/peptidyl |
| Thylakoid membrane protein slr1949 | *slr1949* | 0.77 | 1.11^#^ | 1.33 | Thylakoid membrane protein |
| Uncharacterized thylakoid-associated protein sll0982 | *sll0982* | 2.32 | 0.83 | 1.29^#^ | Thylakoid-associated protein |
| Probable thylakoid lumen protein sll1769 | *sll1769* | 2.25 | 0.99^#^ | 1.19^#^ | Thylakoid lumen protein/peptidyl |
| Thylakoid membrane protein ssl2009 | *ssl2009* | 1.63 | 1.16^#^ | 1.01^#^ | Thylakoid membrane protein |
| Probable thylakoid lumen protein sll0997 | *sll0997* | 1.22^#^ | 0.77 | 0.79^#^ | Thylakoid lumen protein/peptidyl |
| Thylakoid-associated protein slr0729 | *slr0729* | 1.21^#^ | 0.81 | 0.68 | Thylakoid-associated protein |
| Thylakoid membrane protein ssr2422 | *ssr2422* | 1.44 | 0.98^#^ | 0.66 | Thylakoid membrane protein |
| **Cu homeostasis/signal transfer** |  |  |  |  |  |
| Histidine kinase | *slr0222* | 0.98^#^ | 3.04 | 18.53 | Histidine kinase |
| Sensory transduction histidine kinase | *sll1871* | 0.57^#^ | 0.78^#^ | 4.88 | Histidine kinase |
| CheA like protein | *sll0043* | 1.99^#^ | 0.87^#^ | 2.63 | Two-component system |
| Hybrid sensory kinase | *sll1555* | 3.06 | 1.88 | 2.39 | Sensory kinase |
| Sensory transduction histidine kinase | *slr0484* | 0.15 | 0.61 | 0.12 | Histidine kinase |
| **TonB system** |  |  |  |  |  |
| Putative biopolymer transport protein ExbB-like 3 | *sll1404* | 3.00 | 1.92 | 5.32 | Biopolymer transport protein ExbB |
| TonB | *slr1484* | 3.67 | 2.20 | 4.81 | TonB protein |
| Putative biopolymer transport protein ExbD | *sll1405* | 3.02 | 1.86^#^ | 4.07 | Biopolymer transport protein ExbD |
| Putative biopolymer transport protein ExbB-like 1 | *sll0477* | 3.99 | 1.94 | 3.88 | Biopolymer transport protein ExbB |
| Putative biopolymer transport protein ExbD | *slr0678* | 3.56 | 1.11^#^ | 0.26 | Biopolymer transport protein ExbD |
| **Porins** |  |  |  |  |  |
| Sll1550 protein | *sll1550* | 0.97^#^ | 0.75 | 0.68 | Putative porin |
| Slr1908 protein | *slr1908* | 0.83^#^ | 0.78 | 0.60 | Putative porin |
| Slr1841 protein | *slr1841* | 0.75 | 0.79 | 0.50 | Putative porin |
| Sll1271 protein | *sll1271* | 0.84^#^ | 0.86 | 0.49 | Putative porin |
| Slr0042 protein | *slr0042* | 0.79 | 0.83 | 0.47 | Putative porin |
| Sll0772 protein | *sll0772* | 0.51 | 0.55 | 0.31 | Putative porin |
| **Other** |  |  |  |  |  |
| Anti-sigma factor antagonist | *slr1856* | 3.29 | 1.64 | 4.45 | Antagonist |
| Nucleoside diphosphate kinase | *ndk* | 1.65 | 1.61 | 3.75 | Purine metabolism; Pyrimidine metabolism; MAPK (mitogen-activated protein kinase) signaling pathway |
| 16.6 kDa small heat shock protein, molecular chaperon | *hsp17* | 1.3 | 1.16^#^ | 3.58 | Protein processing in endoplasmic reticulum |
| Ribonuclease HI | *rnhA* | 1.9 | 1.24^#^ | 3.13 | DNA replication |
| Cobyrinate a,c-diamide synthase | *cbiA* | 10.13 | 2.53 | 1.83^#^ | Porphyrin and chlorophyll metabolism |
| Extracellular nuclease | *nucH* | 0.47 | 0.88^#^ | 1.77 | Nuclease |
| Probable glutaredoxin ssr2061 | *ssr2061* | 3.01 | 1.11^#^ | 1.66 | Glutaredoxin |
| 1-(5-phosphoribosyl)-5-[(5-phosphoribosylamino) methylideneamino] imidazole-4-carboxamide isomerase | *hisA* | 1.25 | 0.93^#^ | 1.41 | Histidine metabolism |
| Glutamate 5-kinase | *proB* | 3.22 | 1.49 | 0.81^#^ | Carbapenem biosynthesis; Arginine and proline metabolism |
| General secretion pathway protein D | *gspD* | 0.4 | 0.75 | 0.51 | General secretion pathway protein D |
| Twitching mobility protein | *pilT* | 0.3 | 0.7 | 0.49 | Twitching mobility protein |
| Response regulator like protein | *sll1544* | 1.74 | 1.03^#^ | 0.39 | Response regulator like protein |
| Negative aliphatic amidase regulator | *amiC* | 0.28 | 0.69 | 0.34 | ABC transporters |
| Virginiamycin B lyase | *sll0173* | 0.5 | 0.68 | 0.33 | Virginiamycin B lyase |
| Penicillin-binding protein 1B | *mrcA* | 2.24 | 1.56^#^ | 0.33^#^ | Penicillin-binding protein |
| Protein translocase subunit SecD | *secD* | 0.91^#^ | 1.65 | 0.22 | Protein export/Bacterial secretion system |
| Nuclease SbcCD subunit C | *sbcC* | 0.48 | 1.25 | 0.18 | Nuclease |

^#^: *P* > 0.05

**Supplementary Table 3.** Abundance of copper and iron in culture medium and *Synechocystis* sp. PCC 6803 cells. Metal-deduction cells: the *Synechocystis* sp. PCC 6803 cultured for 5 days in BG11 medium lacking Fe and Cu; Medium: BG11 medium lacking Fe and Cu after culturing *Synechocystis* sp. PCC 6803 for 5 days, OD_730_ = 0.685 at 1 cm path length of the ultraviolet spectrophotometer; Normal cells: the *Synechocystis* sp. PCC 6803 cultured in normal BG11 medium, OD_730_ = 0.560 at 1 cm path length of the ultraviolet spectrophotometer.

| **Sample** | **Fe (μmol/L)** | **Cu (nmol/L)** |
| --- | --- | --- |
| Medium | 0.251 ± 0.019 | 17 ± 2 |
| Metal-deduction cells | 8.792 ± 0.216 | 60 ± 2 |
| Normal cells | 64.498 ± 0.384 | 1360 ± 75 |

Metal-deduction cells: the *Synechocystis* sp. PCC 6803 cultured for 5 days in BG11 medium lacking Fe and Cu;

Medium: BG11 medium lacking Fe and Cu after culturing *Synechocystis* sp. PCC 6803 for 5 days, OD_730_ = 0.685 at 1 cm path length of the ultraviolet spectrophotometer.

Normal cells: the *Synechocystis* sp. PCC 6803 cultured in normal BG11 medium, OD_730_ = 0.560 at 1 cm path length of the ultraviolet spectrophotometer.
